# High prevalence of *Angiostrongylus cantonensis* (rat lungworm) on eastern Hawai‘i Island: A closer look at life cycle traits and patterns of infection in wild rats (*Rattus* spp.)

**DOI:** 10.1371/journal.pone.0189458

**Published:** 2017-12-18

**Authors:** Susan I. Jarvi, Stefano Quarta, Steven Jacquier, Kathleen Howe, Deniz Bicakci, Crystal Dasalla, Noelle Lovesy, Kirsten Snook, Robert McHugh, Chris N. Niebuhr

**Affiliations:** 1 Department of Pharmaceutical Sciences, Daniel K. Inouye College of Pharmacy, University of Hawai‘i at Hilo, Hilo, Hawaii, United States of America; 2 USDA-APHIS-WS National Wildlife Research Center, Hawai‘i Field Station, Hilo, Hawaii, United States of America; University of Pretoria, SOUTH AFRICA

## Abstract

The nematode *Angiostrongylus cantonensis* is a zoonotic pathogen and the etiological agent of human angiostrongyliasis or rat lungworm disease. Hawai‘i, particularly east Hawai‘i Island, is the epicenter for angiostrongyliasis in the USA. Rats (*Rattus* spp.) are the definitive hosts while gastropods are intermediate hosts. The main objective of this study was to collect adult *A*. *cantonensis* from wild rats to isolate protein for the development of a blood-based diagnostic, in the process we evaluated the prevalence of infection in wild rats. A total of 545 wild rats were sampled from multiple sites in the South Hilo District of east Hawai‘i Island. Adult male and female *A*. *cantonensis* (3,148) were collected from the hearts and lungs of humanely euthanized *Rattus rattus*, and *R*. *exulans*. Photomicrography and documentation of multiple stages of this parasitic nematode in situ were recorded. A total of 45.5% (197/433) of rats inspected had lung lobe(s) (mostly upper right) which appeared granular indicating this lobe may serve as a filter for worm passage to the rest of the lung. Across *Rattus* spp., 72.7% (396/545) were infected with adult worms, but 93.9% (512/545) of the rats were positive for *A*. *cantonensis* infection based on presence of live adult worms, encysted adult worms, L3 larvae and/or by PCR analysis of brain tissue. In *R*. *rattus* we observed an inverse correlation with increased body mass and infection level of adult worms, and a direct correlation between body mass and encysted adult worms in the lung tissue, indicating that larger (older) rats may have developed a means of clearing infections or regulating the worm burden upon reinfection. The exceptionally high prevalence of *A*. *cantonensis* infection in *Rattus* spp. in east Hawai‘i Island is cause for concern and indicates the potential for human infection with this emerging zoonosis is greater than previously thought.

## Introduction

The rat lungworm (*Angiostrongylus cantonensis*) is a nematode that causes human angiostrongyliasis (frequently called rat lungworm disease or RLWD). East Hawai‘i Island is the epicenter for angiostrongyliasis in the USA [1]. Symptoms of *A*. *cantonensis* infection range from mild (flu-like) to severe (including eosinophilic meningitis, paralysis, and coma), and the infection can be fatal in humans [2, 3]. Rats (*Rattus* spp.) are the definitive hosts while gastropods are obligatory intermediate hosts. *Angiostrongylus cantonensis* was initially discovered in China [4], and in Hawai‘i the first human case of angiostrongyliasis was reported in 1959 [5]. This zoonotic pathogen is also found in Asia, Australia, Brazil, the Caribbean islands and other Pacific Islands and has spread to and within the US continent (Louisiana, Texas, Oklahoma, and Florida) with more than 2,800 cases of human infection reported in 30 countries [2, 6, 7, 8]. *Angiostrongylus cantonensis* is typically described as a tropical parasite but it appears to be adapting to gastropod hosts found in more temperate climates [9]. The number of reported human angiostrongyliasis cases has recently increased in Hawai‘i and this increase parallels the circa 2004 introduction and subsequent spread of the semi-slug (*Parmarion martensi*) in east Hawai‘i Island [10]. For the period 2010–2014 an average of 2.4 human cases per year were reported by the Hawai‘i Health Information Corporation (HHIC), which bases numbers on discharge data from hospitals and emergency rooms statewide, indicating that the cases were sufficiently severe to warrant a trip to the hospital. In 2015 the HHIC reported nine cases, in 2016 reported 21 cases, and from January to April 2017, reported 16 cases of human angiostrongyliasis in Hawai‘i, including an outbreak on Maui. An invasive, highly competent intermediate host, *P*. *martensi*, has also recently been documented on Maui by one of the authors of this paper (Kay Howe, personal communication). Based on these data there is an obvious, relatively recent, upward trend in the number of cases of human angiostrongyliasis, most of them originating on Hawai‘i Island.

Three rat species are found in Hawai‘i, including the Polynesian rat (*R*. *exulans*), the Norway rat (*R*. *norvegicus*), and the black rat (*R*. *rattus*). The arrival of Polynesian rats in Hawai‘i is attributed to voyaging Polynesians, estimated between 1219–1266 A.D [11]. Norway rats are thought to have arrived in Hawai‘i between 1825–1842 [12, 13], while black rats are thought to have arrived on Oahu, Hawai‘i between 1870–1890 and subsequently spread to the other Hawaiian Islands over the next 10–15 years [14]. The Norway rat is the largest rat found in Hawai‘i, reaching 203 to 254 mm long. The black rat is a medium to large sized rat (127 to 178 mm long) with the Polynesian rat being smaller in size (102 to 127 mm long).

In a previous study [15], all three species of rats were captured in 2009 and 2011 at six different locations on east Hawai‘i Island, 54% (20/37) tested positive for adult *A*. *cantonensis* worms with 1 to 30 adult worms detected in each positive rat. In this study, 100% of rat lung samples tested positive by real-time PCR for *A*. *cantonensis*, including those that were not visually positive for adult worms. In another previous study [1] 61 rats (*R*. *rattus*) were trapped in the Waiakea Forest Reserve in east Hawai‘i Island at 533 m elevation. Upon necropsy 10/61 (16.4%) of rats examined had adult worms in their hearts and/or lungs, with a range of 1–24 worms and with an average of 7.5 adult worms found. Surveys of *A*. *cantonensis* infection in other *R*. *rattus* studies show similar levels in Jamaica (12.9%), Australia (16%), Puerto Rico (15.9%), and the Canary Islands (15%) at one time point and much higher (55.6%) at another [16]. A recent study conducted in Florida found 22.8% (39/171) *R*. *rattus* positive for *A*. *cantonensis* [9].

The life cycle of *A*. *cantonensis* includes five larval stages (L1-L5) before becoming a sexually mature adult with larvae increasing in size throughout each stage of growth [17]. Larval stages L1 through L3 develop in the intermediate gastropod host. L1 larvae measure approximately 0.3 mm in length and 0.015 mm in width. Sufficient growth to reach the threshold of L2 occurs within six-twelve days and molting begins. L2 larvae are about 0.45 by 0.03 mm with L3 larvae being of similar size. It is the L3 larvae which are infective to rats, humans and other susceptible hosts. In humans, ingestion of the L3 larvae on uncooked or unfrozen produce or in liquids (e.g. water) are thought to be the main causes of infection. Rats become infected by consuming an infected gastropod harboring L3 larvae. L3 larvae then burrow into the small intestine and enter the rat’s bloodstream. L3 larvae are carried in the blood to the brain, penetrating the central nervous system (CNS) within 24 hours. The larvae continue to grow within the brain. During the sixth or seventh day after ingestion, molting occurs again. The larvae at this point are now L4 stage with a length around 1.0 mm by 0.04 mm in width; by the time they become young sub adults (L5), they are approximately 2 mm by 0.06 mm in diameter. Before leaving the brain the L5 larvae grow to approximately 12 mm (females) and 11 mm (males) in length. L5 larvae departing the brain are carried by the rat host’s blood flow to the heart and lungs, where, if successful, they may ensconce in a chamber of the heart or in the lung tissue, but with a majority ultimately residing in the pulmonary artery. These adult worms reside in the heart and lungs and increase substantially in size (females 35 mm x 0.6 mm, males 25 mm x 0.4 mm), become sexually mature and reproduce thus completing the life cycle [17].

In this study we visually document multiple stages and locations of development for *A*. *cantonensi*s in wild rats, determine prevalence, and examine patterns of infection. We describe incidental evidence of the impact of infection on rat tissues, and evaluate data trends of *A*. *cantonensis* infection in wild-caught rats from east Hawai‘i Island. We then interpret and discuss how these data can be used to improve risk assessment and guide research development to better prevent and control human infection with *A*. *cantonensis* in affected areas.

## Methods

### Study area

**Fig 1:**
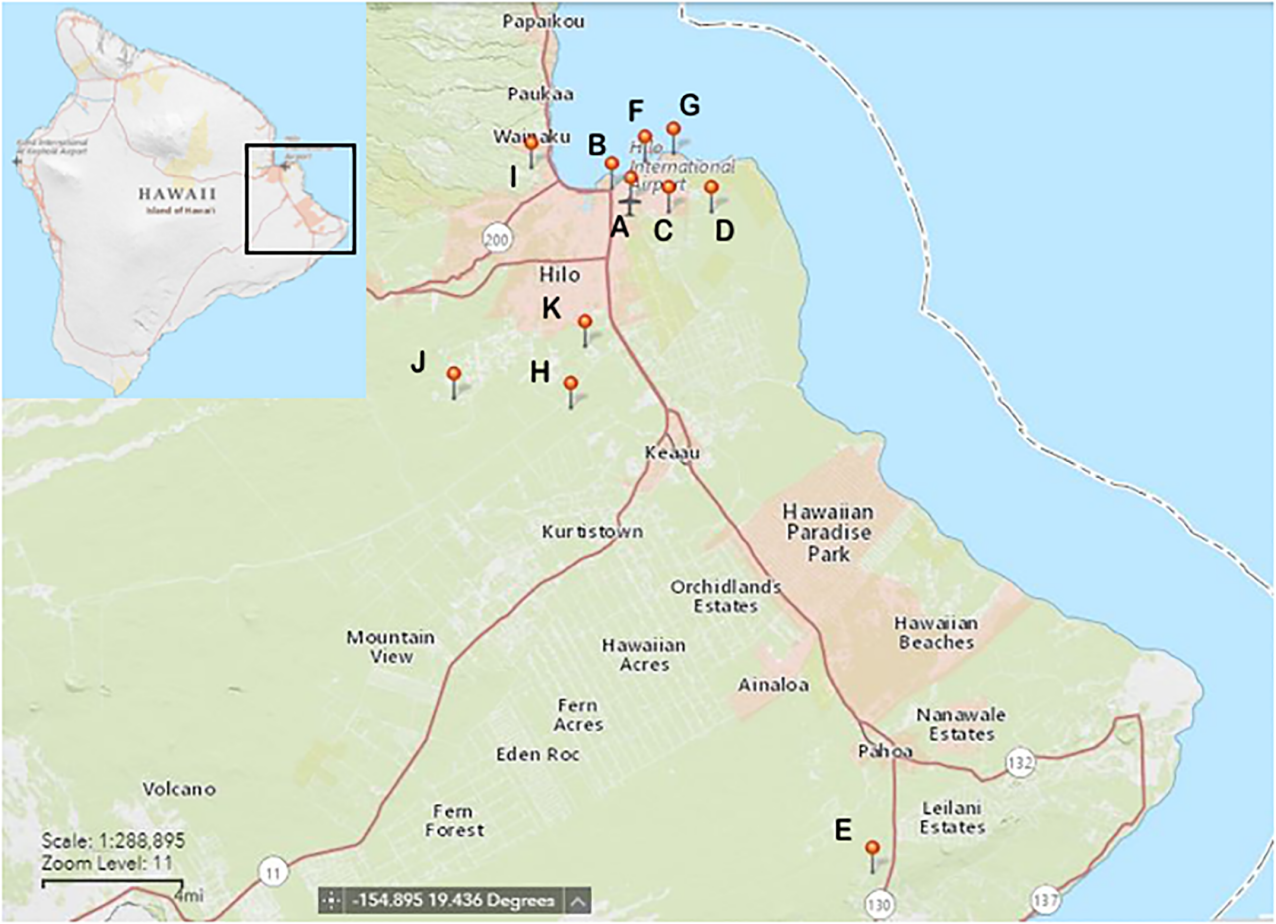
Map of trapping sites in east Hawai‘i Island. Refer to Table 1 for details of all site locations.

Sampling was conducted between 31 January and 24 February 2017 on the east side of Hawai‘i Island, in and around the South Hilo District (Fig 1). This region has a tropical rainforest climate with considerable rainfall occurring throughout the year (average of 3218 mm) with an average annual temperature of 23.25°C.

### Rat capture

Wild rats were captured from multiple sites in the South Hilo District of east Hawai‘i Island by USDA-APHIS National Wildlife Research Center (NWRC) and the Hawai‘i Department of Health (HDOH) Vector Control Branch. Due to the paucity of data on *A*. *cantonensis* infection in rats in Hawai‘i, rat trapping was conducted opportunistically. Sampling sites chosen and number of traps used were based on minimizing trapping effort and maximizing individuals caught. Trapping efforts ranged from sea level to 400 m in elevation and typically consisted of sites along roads and forest-edge in close proximity to urban areas, along with one rural farm site. No specific permission for trapping was required from the authorities, because the target species are classified as invasive pests and the methods used did not involve any endangered or protected species. However, permission for trapping was given by the landowners. Wild rats were trapped using Haguruma live-traps (Osaka, Japan), and transported to a laboratory facility in early morning. Rats were humanely euthanized in a CO_2_ chamber upon arrival to the lab. Every effort was made to ensure minimal suffering. Rats were immediately dusted with pyrethrin post-euthanisia. All animal procedures were conducted according to the Guidelines of the American Society of Mammalogists for the use of wild mammals in research [18] and following approved Institutional Animal Care and Use Committee protocols (US Department of Agriculture, National Wildlife Research Center) QA-2747 and (University of Hawaii) 17–2553. This study was approved by University of Hawaii Institutional Biosafety Committee (protocol 17-01-505-08A).

### Harvesting of worms

Post euthanasia, rats were assigned a specimen ID number and the species, body mass, and sex (when discernable) was recorded. The heart and lungs were removed from each specimen and placed in a standard petri dish. Using either Leica or Olympus (4X-10X) dissecting microscopes, adult worms were manually extracted with fine-tipped forceps from the pulmonary artery, heart, and lungs. Other data collection (e.g. presence of L3 larvae, encysted adults, and a recognizable pattern of granular progression in one to five lung lobes) was based on observations as the study progressed; these sometimes did not occur or were not detected until part way through the study. Thus, recording for some measures was not uniform and complete throughout from start to finish in all specimens. Fresh rat feces from some rats were collected from under the rat traps and immediately dissolved in tap water for microscopic viewing. A main goal of this study was to isolate adult *A*. *cantonensis* for diagnostics development and exposure to bleach would cause deleterious protein degradation. Consequently, dissection tools were washed between uses on each rat in 70% ETOH instead of bleach and wiped dry with disposable Kimwipes^®^ (Kimberly-Clark, Roswell, GA, USA) between uses. All tools were soaked and cleaned in bleach at the end of each day. Harvested worms were tallied by sex, washed 3X in 1X PBS and placed in pre-weighed tubes containing cold 1X PBS with 1X protease inhibitor (Halt^™^ Protease and Phosphatase Inhibitor Single-Use Cocktail EDTA-free, Thermo Fisher Scientific, Waltham, MA, USA) on ice. Worms found within sections of heart/lung contaminated with rat hair and/or pyrethrin powder as well as worms that burst upon isolation were discarded and were not tallied. Whenever a tube became at least half full, that tube was again weighed to determine the net mass of the worms, and then stored at -80°C. A sample of the heart/lung tissue for every rat dissected was placed in 150 ul DNA lysis buffer (0.1 M Tris–HCl, 0.1 M EDTA, 2% SDS) and stored at -80°C. The rat carcasses and compartmentalized heart/lung tissues were placed in an individually labelled plastic bag and frozen (immediately at -20°C and then moved to -80°C for long term storage). Prior to carcass disposal, rat heads were collected from 273 carcasses and stored along with the corresponding heart and lung tissue at -80°C. Images and video taken throughout were captured by a MiniVID microscope digital camera (LW Scientific, Inc., Lawrenceville, GA, USA), and processed by TOUPVIEW software v. 3.7 (AmScope, Irvine, CA, USA).

### Molecular detection of *A*. *cantonensis*

DNA was extracted and qPCR carried out as previously described [19] on extracted brain tissues, several samples of microscopically small (partially) black worms observed in the lungs, and other suspected non-*A*. *cantonensis* worms. The brains from rats that were either completely negative for all visual cues of infection (n = 27) or were negative for all visual cues but were not completely evaluated (n = 11) were dissected for the visual and molecular detection of *A cantonensis* (29 *R rattus*; 7 *R exulans*; 2 unknown). An approximate 30 mg cross-section of the cerebellum, taken diagonally from the superior-anterior to the inferior-posterior, which included white matter from the brain stem, was the standard tissue collected and stored in DNA lysis buffer or ATL (Qiagen, Valencia, CA, USA). Dissection tools were either individual use (blades), or cleaned with bleach, rinsed 2X with water, dipped in 70% ETOH to dry, and exposed to 200 m J/cm^2^ for 1 minute on each side in a UVP cx-2000 UV crosslinker between uses. If worms were found anywhere in the brain, they were processed separately for verification of species or were combined with a portion of adjacent brain tissue.

### Data analysis

Generalized linear models (GLMs) were used to explore the effects of host species, host sex, host body mass, and sampling site on infection levels of *A*. *cantonensis*. Rats were grouped by body mass into six categories (<45, 45–60, 60–90, 90–120, 120–150, and ≥150 g). If an individual’s body mass was between categories, then it was grouped with the upper category. A binary logistic regression was used to model the prevalence (herein defined as presence/absence observed in this study) of live adult *A*. *cantonensis*, encysted adult worms, L3 larvae, and granular lung lobe(s), as well as a negative binomial distribution with log link to investigate adult worm intensity (mean number of worms present in infected rats). Due to incomplete values for some sampled rats, only individuals with recorded data from each of the four variables were included in the analysis. We included all combinations and interactions of predictors and then used a model selection approach to identify best-fit models using Akaike’s Information Criterion corrected for small sample size (AICc). Models were ranked based on the lowest AICc value, with models differing by two units considered competitive (ΔAICc ≤ 2). Chi-square tests were used to compare rates of infection and unpaired, two-tailed *t*-tests were used to compare mean body masses. All analyses were performed using the statistical software IBM SPSS Statistics version 20.0 (SPSS Inc. Chicago, IL, USA) and a significance level of 0.05 was used.

## Results

### Data summary

A total of 545 *Rattus* spp. were sampled from 11 sites from the east side of Hawai‘i Island and examined for *A*. *cantonensis* infection (Table 1, Fig 1). The number of rats trapped differed among sites due to the varying trapping efforts and opportunistic sampling methods. Due to incomplete data, sample sizes differ between total rats sampled (n = 545), rats of known species (n = 510), and rats of known sex (n = 409). Two rat species were trapped in this study, *R*. *exulans* (200/510: 39.2%) and *R*. *rattus* (310/510: 60.8%), with slightly more female rats (239/409: 58.4%) than males (170/409: 41.6%). The mean body masses for *R*. *exulans* and *R*. *rattus* in this study were 48.0 g and 96.8 g, with the largest individuals weighing 88.0 g and 241.8 g respectively.

**Table 1:** List of trapping locations for rats (*Rattus*. *exulans* and *Rattus rattus*) sampled for rat lungworm (*Angiostrongylus cantonensis*) on east Hawai‘i Island (see Fig 1 for site map). Values for infected rats indicate individuals observed positive for live adult worms only. Agencies involved in trapping include USDA-APHIS National Wildlife Research Center (NWRC) and the Hawai‘i Department of Health (HDOH) Vector Control Branch.

Site	Agency	# of rats sampled	# infected rats	Latitude (°N)	Longitude (°W)	Elevation (m)
A	NWRC	227	177 (78%)	19.714	-155.055	10
B	NWRC	30	26 (87%)	19.720	-155.063	6
C	HDOH	17	11 (65%)	19.710	-155.038	12
D	HDOH	3	2 (67%)	19.710	-155.02	10
E	NWRC	16	10 (63%)	19.440	-154.95	311
F	HDOH	19	5 (26%)	19.731	-155.049	1
G	HDOH	10	8 (80%)	19.734	-155.036	1
H	NWRC	35	25 (71%)	19.630	-155.081	232
I	NWRC	3	0 (0%)	19.728	-155.098	71
J	NWRC	27	9 (33%)	19.634	-155.132	400
K	NWRC	158	123 (78%)	19.655	-155.075	115
**Total**		**545**	**396 (72.7%)**			

**Fig 2:**
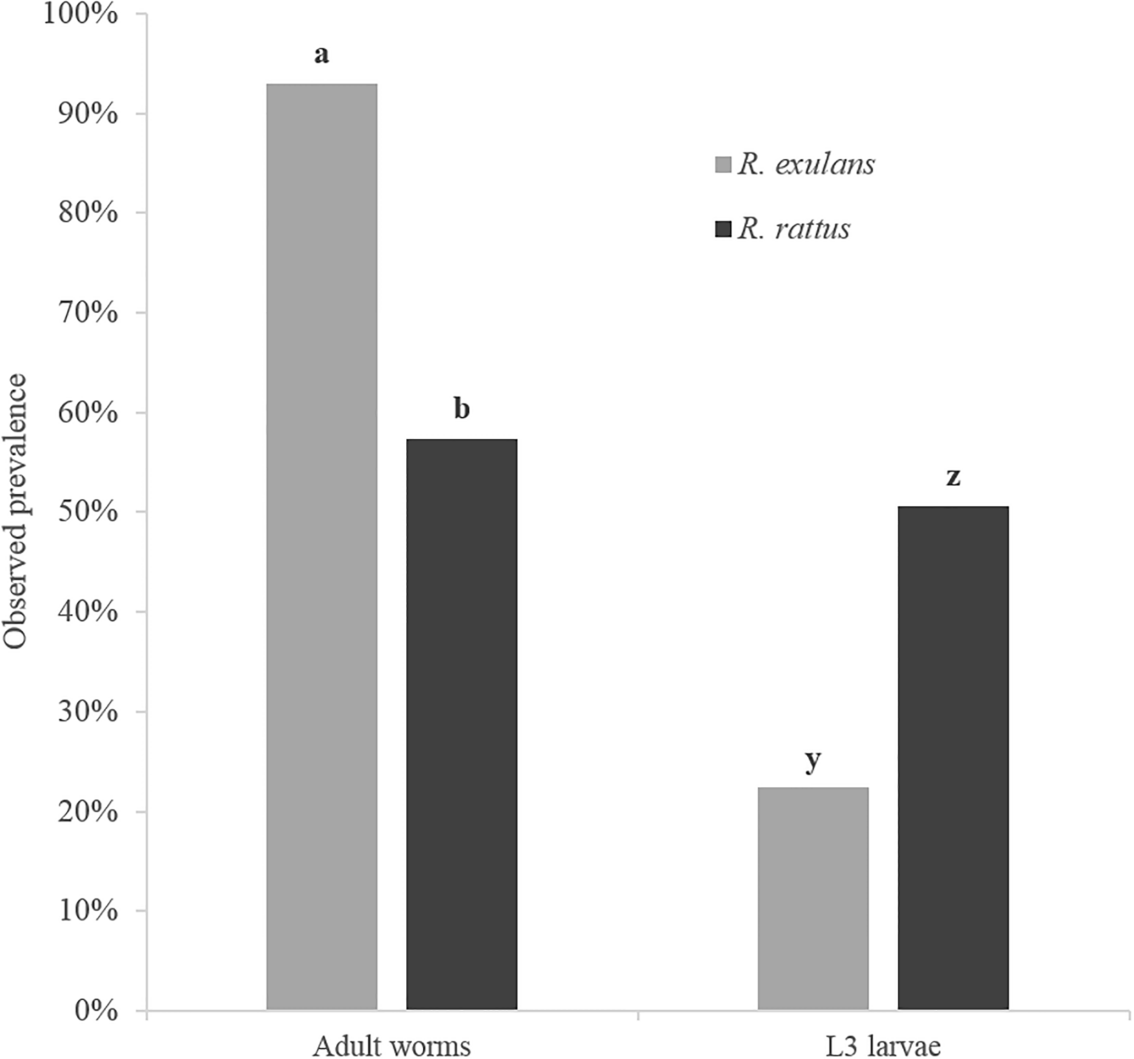
Observed prevalence of adult and third stage (L3) larvae of rat lungworms (*A*. *cantonensis*) in the lungs of two species of wild rats (*R*. *exulans* and *R*. *rattus*) on east Hawai‘i Island. Columns within groups with different letters differ (P < 0.05).

Overall, 72.7% (396/545) of *Rattus* spp. were infected with live adult stages of *A*. *cantonensis*. We observed a higher rate of infection of adult worms in *R*. *exulans* (93.0%; 186/200) compared to *R*. *rattus* (57.4%; 178/310) (χ^2^ = 75.32, d.f. = 1, P < 0.0001) (Fig 2). The mean body mass of *R*. *rattus* individuals infected with adult worms (79.1 ± 5.9 g; P < 0.0001), was significantly lower than uninfected individuals (120.8 ± 6.7 g) with no difference observed in *R*. *exulans* (Table 2). A total of 3,148 adult worms were harvested with an observed mean intensity of 8.01 (n = 396). A number of worms were lost to contamination (hair or pyrethrin) and/or excessively damaged during extraction and so were not included in the study. Mean intensities of adult *A*. *cantonensis* observed in infected *R*. *exulans* and *R*. *rattus* were 8.97 and 7.09 respectively, while mean intensities of female (n = 153) and male (n = 123) *Rattus* spp. individuals were 7.88 and 8.16, respectively. The greatest number of adult worms harvested from a single individual rat was 45 from *R*. *rattus* (host body mass of 66.3 g), with 31 adult worms found in a single *R*. *exulans* individual (host body mass of 45.0 g).

**Table 2:** Mean body mass (g) and confidence intervals for observed presence/absence of adult rat lungworms (*Angiostrongylus cantonensis*), third stage (L3) larvae, encysted adult worms, and granular lung lobes in wild rats (*Rattus* spp.) on east Hawai‘i Island. All p-values reported are from comparisons of body mass between positive and negative individuals within each group using unpaired, two-tailed *t*-tests.

Observed	*R*. *exulans*			*R*. *rattus*		
	Positive	Negative	p-Value	Positive	Negative	p-Value
Adult worms	48.5 ± 1.8	42.1 ± 8.3	> 0.05	79.1 ± 5.9	120.8 ± 6.7	< 0.0001
L3 larvae	50.3 ± 4.5	46.5 ± 2.0	> 0.05	107.1 ± 6.6	90.1 ± 8.4	< 0.01
Encysted worms	60.0 ± 5.4	46.9 ± 1.8	< 0.0001	114.6 ± 8.6	90.6 ± 5.8	< 0.0001
Granular lungs	48.9 ± 2.1	47.3 ± 3.7	> 0.05	68.1 ± 7.8	104.5 ± 6.4	< 0.0001

**Fig 3:**
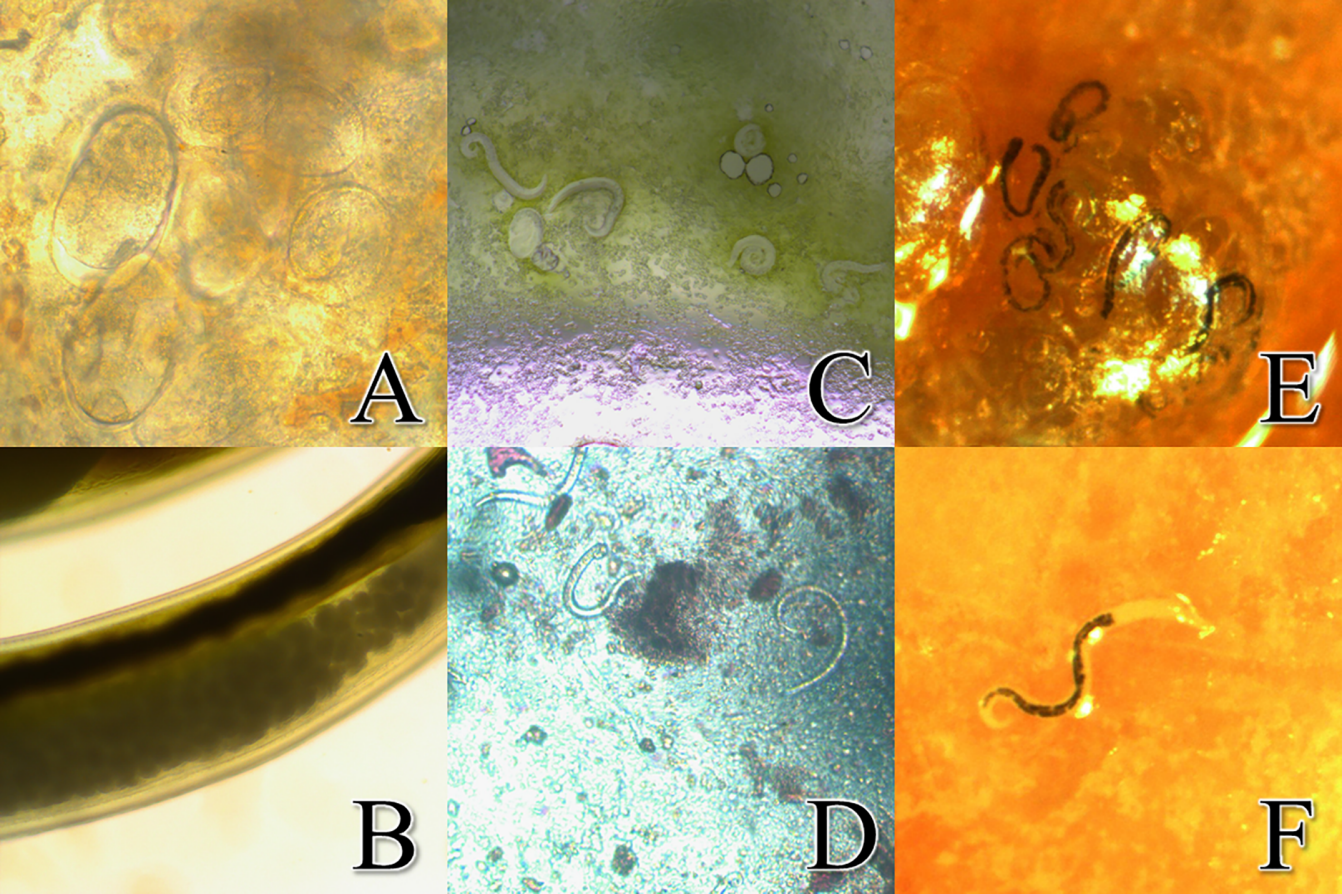
Images of multiple stages of *Angiostrongylus cantonensis* in situ. *a*. Embryonated *A*. *cantonensis* eggs in the lung tissue of a rat (40X). *b*. *A*. *cantonensis* eggs that were located in the uterus of a female worm (40X). *c*. *A*. *cantonensis* L1 larvae in lung visualized by tissue squash (10X). *d*. *A*. *cantonensis* L1 larvae observed in rat feces (10x). *e*. Small black worms, later determined to be L3 *A*. *cantonensis* larvae observed in the rat’s lungs. *f*. The transition between the esophagus and intestine are clearly defined in *A*. *cantonensis* L3 larvae.

**Fig 4:**
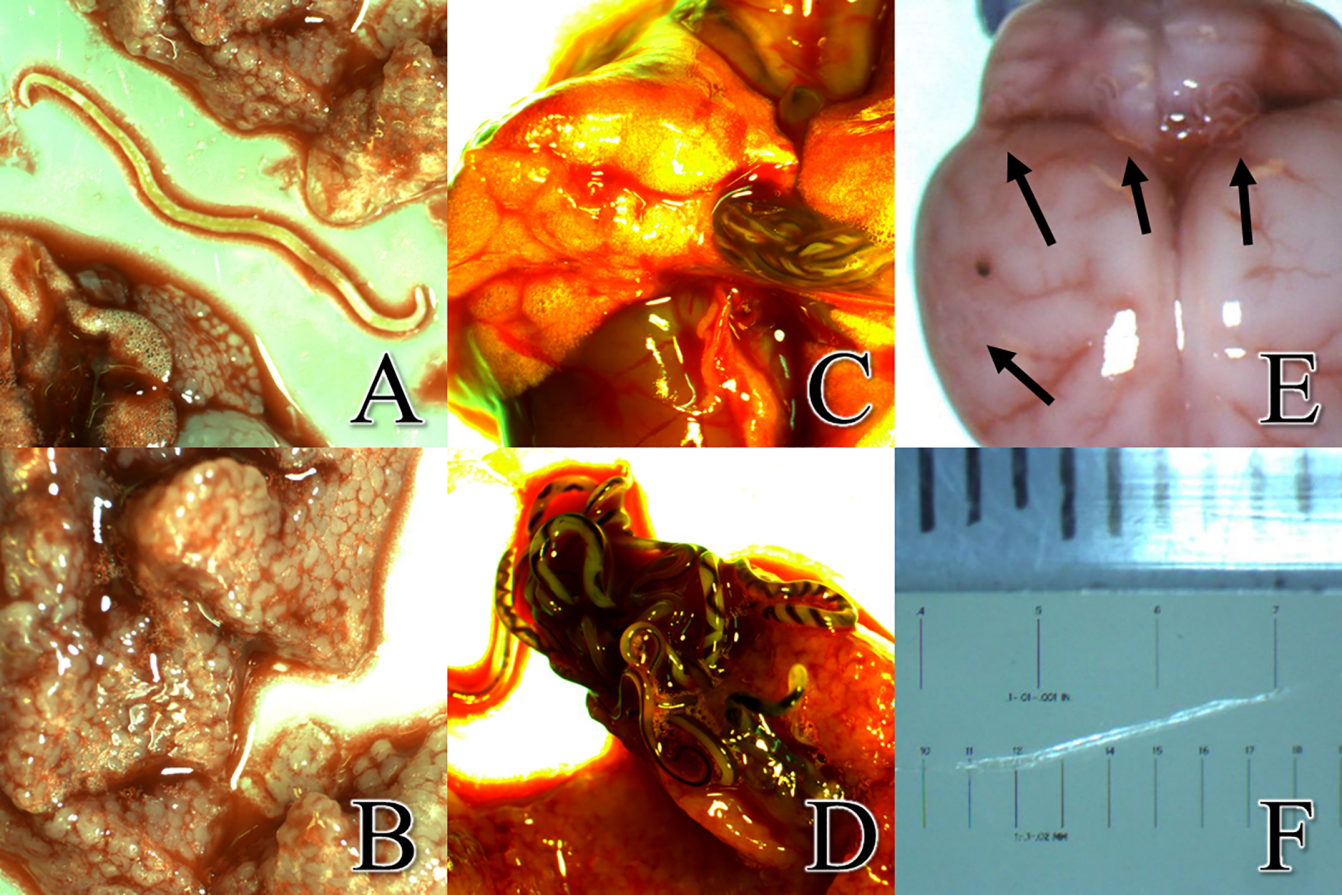
Evidence of infection included presence of decaying encysted adults, granular lung lobes, live adult worms in the pulmonary artery and detection of worms in the brain. *a*. Decaying, encysted adult *Angiostrongylus cantonensis* worms, apparently indicative of a previous infection, were observed in the rat’s lungs. *b*. Granular upper right lobe of lung. *c*. Adult *A*. *cantonensis* visible in the intact pulmonary artery. *d*. Adult *A*. *cantonensis* emerging from the pulmonary artery of a rat. Males are smaller and females are larger with helical-striped appearance. (See S1 Video). *e*. Superior view of *Rattus exulans* brain at a magnification of 6.6X. During dissection, *A*. *cantonensis* were commonly found at locations indicated by arrows. The dark spot seen on the surface of the right hemisphere is believed to be a hemorrhage. Hemorrhages were often times an indicator of worm location during dissections. *f*. Measurement of *A*. *cantonensis* L5 from the brain measuring approximately 10 mm. The exsiccated nature of the worm is due to light intensity of the microscope.

The 3,148 adult *A*. *cantonensis* worms had a total mass of approximately 20 grams. Most of the worms were found in, or closely associated with, the pulmonary artery, with some adult worms found in the heart or lung tissue itself. Images of *A*. *cantonensis* eggs were captured in both rat lung tissue (Fig 3a) and the uterus of a gravid *A*. *cantonensis* female (Fig 3b). Microscopic L1 larvae were observed in the rat lungs (Fig 3c) and in the feces (Fig 3d). Substantially larger yet still microscopic small black worms were observed in the lung tissue of 39% (168/427) of individuals (Fig 3e and 3f). We observed a lower rate of infection of L3 larvae in *R*. *exulans* (22.4%; 38/170) compared to *R*. *rattus* (50.6%; 130/257) (χ^2^ = 34.17, d.f. = 1, P < 0.0001) (Fig 2). The mean body mass of *R*. *rattus* individuals infected with L3 larvae (107.1 ± 6.6 g; P < 0.01) was significantly higher than uninfected individuals (90.1 ± 8.4 g), with no difference observed in *R*. *exulans* (Table 2). The DNA of multiple small black worms was extracted from five specimens, and subjected to PCR analysis which confirmed them as *A*. *cantonensis*. Since these worms were observed in the lung tissue, they can only be newly hatched L1 larvae or a current infection of L3 larvae, since L2 larvae would not survive the acidic conditions in the rat stomach [17]. Based on size and movement [20], in combination with PCR results, the small black worms were thus determined to be L3 *A*. *cantonensis* larvae. Also noted in 18% (98/545) of rats was the presence of decaying and encysted adult *A*. *cantonensis* in the lungs (Fig 4a), possibly a definitive sign of an older infection. For both *R*. *exulans* and *R*. *rattus*, the mean body mass for individuals observed with encysted worms was higher than those observed without (60.0 ± 5.4 vs 46.9 ± 1.8 and 114.6 ± 8.6 vs 90.6 ± 5.8 g respectively; P < 0.0001) (Table 2). In total, 90% (490/545) of rats sampled in this study were visually positive for *A*. *cantonensis*, through observation or collection of live adult worms, encysted adults, or L3 larvae. During the study, we documented rats with granular lobes of the lungs, most commonly observed in the upper right lobe (Fig 4b). The tissue characteristics and apparent respiratory viability of the lungs’ lobes varied considerably, as previously documented by [17]. We observed granular lungs in 45% (197/433) of all rats inspected, and in 60% (177/315) of rats infected with adult worms. Of note, 100% (197/197) of all rats showing signs of granular lung lobes were positive for adult worms. The mean body mass of *R*. *rattus* individuals with granular lung lobes (68.1 ± 7.8 g) was significantly lower than uninfected individuals (104.5 ± 6.4; P < 0.0001), with no difference observed in *R*. *exulans* (Table 2). An image of the abundance of worms packed into the pulmonary artery of a rat is visible in Fig 4c, and adult worms emerging from a pulmonary artery which has been ruptured is shown in Fig 4d. Of note, a large abdominal burden from one type of intestinal worm, pending identification and presumed to be the common acanthacephalan (*Moniliformis moniliformis*), was present in large quantity in both number of individuals and in biomass in one individual.

The skulls of 38 rats were dissected and visually 24% (9/38) of rats had worms on the surface of the brain. Of those visually positive 77.8% (7/9) larvae were primarily found at the superior-anterior position of the cerebellum, along the cerebral transverse fissure (Fig 4e). Of the worms visualized, most worms appeared to be L3 larvae based on size, but in 33.3% (3/9) the worms were larger; these worms were presumed to be at the L5 stage and measured ~10 mm in length (Fig 4f). A total of 60.5% (23/38) of brains had discoloration on their surface (dot in left side of Fig 4e). Qualitative PCR resulted in 22 samples testing positive for *A*. *cantonensis*, seven samples testing negative and nine samples were undetermined. Of the samples visually positive for worms, 100% (9/9) came up positive by PCR and also had discoloration on the surface of the brain. All *R exulans* tested positive (7/7) by PCR. Based on data including presence of live adult worms, encysted adult worms, L3 larvae, and/or were positive by PCR analysis of brain tissue, a total of 90% (280/310) of *R*. *rattus* were infected, and 99%, (198/200) *R exulans* were infected with *A*. *cantonensis*. Among all 545 rats captured, 93.9% (512/545) were positive for *A*. *cantonensis* infection.

### Model factors influencing *A*. *cantonensis* infection

**Fig 5:**
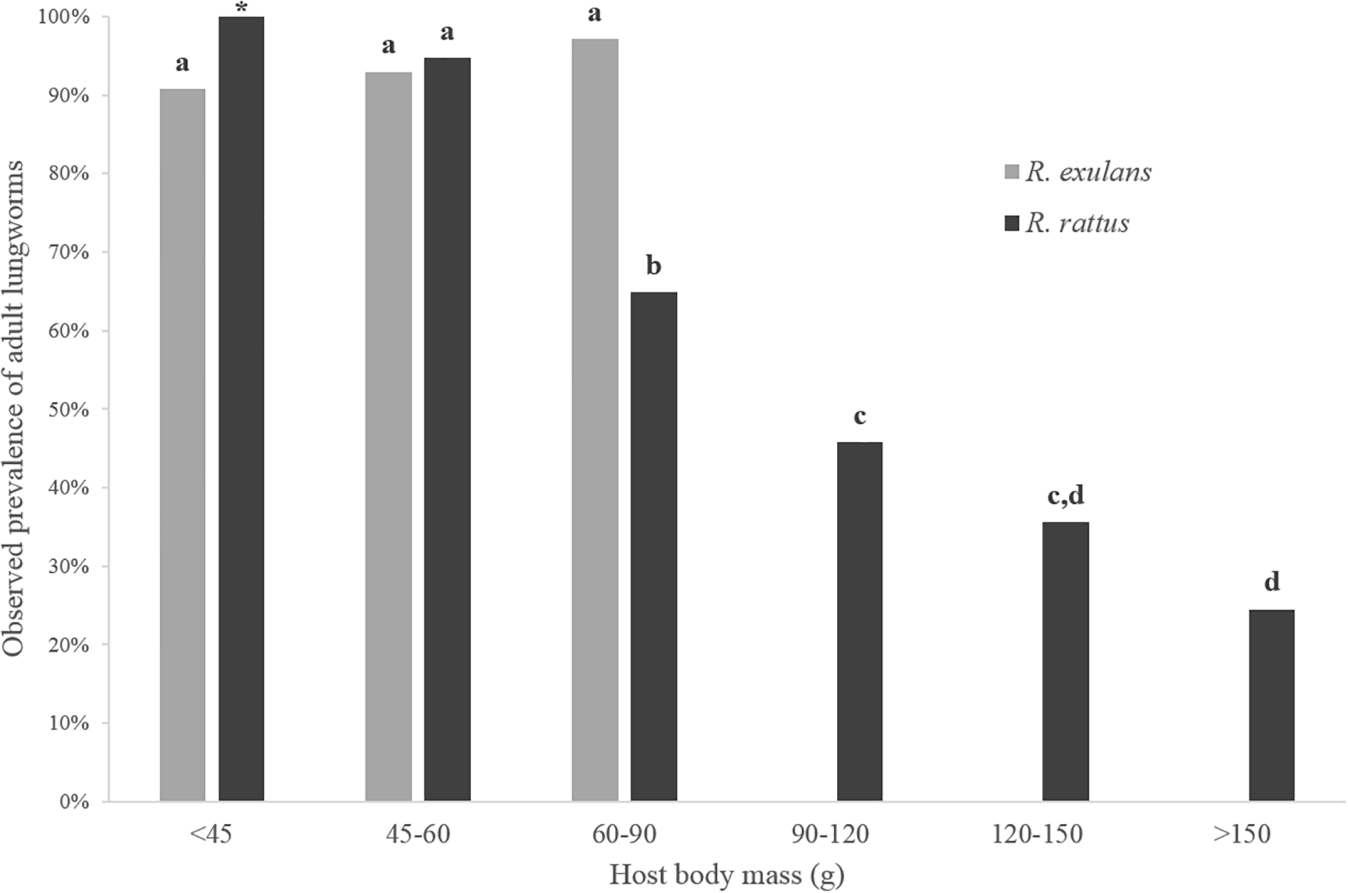
Observed prevalence of adult rat lungworms (*Angiostrongylus cantonensis*) for multiple body mass categories in two species of wild rats (*Rattus exulans* and *R*. *rattus*) on east Hawai‘i Island. Results displayed are from rat individuals used in the GLM analysis only (n = 405; see Table 2). Columns with different letters differ (P < 0.05). The star indicates significance could not be determined due to low sample size.

Results from the five GLM analyses resulted in multiple candidate models (Table 3A–3E), with the best-fit models discussed here. Host species and body mass factors contributed to the observed prevalence of adult *A*. *cantonensis* (Wald χ^2^_8_ = 92.142, P < 0.001) (Table 3A), with more infected *R*. *exulans* 92.9% (n = 168) identified than *R*. *rattus* 49.8% (n = 237). These values were similar to those observed in the overall summary statistics from this study (Fig 2). However, an interaction effect between host species and body mass was identified as an inverse correlation observed between adult worm prevalence and host body mass, but only for *R*. *rattus* (Fig 5). Host body mass also contributed to *A*. *cantonensis* infection intensity in *Rattus* spp. (Wald χ^2^_5_ = 19.156, P = 0.002) (model 1; Table 3B), with mean adult worm numbers observed at 8.2 (n = 84), 9.6 (n = 76), 8.7 (n = 92), 4.2 (n = 59), 4.5 (n = 45), and 4.2 (n = 49) respectively from lowest to highest body mass category. As seen with live adult worm prevalence, host body mass was also significantly associated with the presence of encysted worms (Wald χ^2^_5_ = 41.343, P < 0.001) (Table 3C), but with an opposite, direct correlation observed. Prevalence of encysted worms was observed at 4.8% (n = 84), 9.2% (n = 76), 28.3% (n = 92), 35.6% (n = 59), 40.0% (n = 45), and 36.7% (n = 49) respectively from lowest to highest body mass category. The observed prevalence of L3 larvae was best explained by host species (Wald χ^2^_1_ = 43.706, P < 0.001) as well as sampling site (Wald χ^2^_10_ = 39.974, P < 0.001), but not body mass (Table 3D). Prevalence of L3 larvae was higher in *R*. *rattus* 55.3% (n = 206) than *R*. *exulans* 22.9% (n = 33), values which were similar to those observed in the overall summary statistics from this study (Fig 2). As seen in the adult worm prevalence analysis, we identified a significant interaction between host species and body mass using prevalence of granular lungs as the response variable (Wald χ^2^_8_ = 71.045, P < 0.001) (Table 3E). However while the presence of granular lungs did not differ significantly between body mass categories for *R*. *exulans*, differences were observed for *R*. *rattus*, supporting the trend seen in Table 2.

**Table 3:** Summary of AICc (Akaike’s Information Criterion corrected for small sample size) model selection of rat lungworm (*Angiostrongylus cantonensis*) infection in rats (*Rattus* spp.) sampled on east Hawai‘i Island for five independent generalized linear model (GLM) analyses (A-E). (A) Prevalence of adult worms, (B) intensity of adult worms, (C) prevalence of encysted worms, (D) prevalence of third stage larvae in the lungs, (E) prevalence of granular lung lobes. Model factors include host characteristics (body mass, species, and sex) and sampling location (site); numbers preceding models indicate model rank within each analysis; k = number of parameters in the models.

Analysis	Model	k	Log-Likelihood	AICc	ΔAICc
**(A) Prevalence of adult worms**	1. Mass*Species^†^	7	-90.889	200.23	0.00
	2. Mass*Species^†^ + Sex	8	-90.120	200.24	0.01
**(B) Intensity of adult worms**	1. Mass^†^	6	-843.809	1699.94	0.00
	2. Sex*Species^†^	3	-846.450	1701.05	1.11
	3. Species + Mass	7	-843.328	1701.08	1.14
	4. Species^†^	2	-848.617	1701.28	1.34
**(C) Prevalence of encysted worms**	1. Mass^†^ + Site	16	-80.695	194.79	0.00
	2. Mass*Species^†^ + Site	17	-77.899	195.77	0.98
	3. Mass^†^ + Site + Species	17	-80.461	196.50	1.71
**(D) Prevalence of 3rd stage (L3) larvae**	1. Species^†^ + Site^†^	12	-83.916	192.76	0.00
	2. Species^†^ + Site^†^ + Sex	13	-83.472	192.95	0.19
	3. Sex*Species^†^ + Site^†^	13	-82.029	193.31	0.55
	4. Species^†^ + Site^†^ + Mass	17	-79.074	193.99	1.23
	5. Species^†^ + Site^†^ + Sex + Mass	18	-78.128	194.33	1.57
	6. Sex*Species^†^ + Site^†^ + Mass	18	-77.020	194.34	1.58
**(E) Prevalence of granulated lobes**	1. Mass*Species^†^ + Site	17	-76.783	193.862	0.00
	2. Mass*Species^†^ + Site + Sex	18	-76.638	195.820	1.96

^†^ Indicates a significant variable or interaction in the model (P < 0.05); asterisks (*) denote interaction terms.

## Discussion

Eastern Hawai‘i Island appears to have an extremely high prevalence of *A*. *cantonensis* infection in *Rattus* spp. and is the epicenter for angiostrongyliasis in the USA. It is known that the parasite exists on all main Hawaiian Islands in a variety of intermediate gastropod host species [21]. It is also noted that the increase in the number of human angiostrongyliasis cases has paralleled the introduction of the semi-slug *P*. *martensi* to east Hawai‘i, and that the semi-slug is also heavily infected [10]. A previous study was completed on Hawai‘i Island examining the definitive hosts in which 37 rats from east Hawai‘i Island were evaluated morphologically and by real-time PCR for *A*. *cantonensis* infection levels [15]. Most of the rats in this prior study were captured in 2009 and 2011 in the Puna district, which is adjacent to the South Hilo district, and while only 54% were visually positive for adult worms, 100% were positive for *A*. *cantonensis* by PCR. This demonstrates either current or prior infection in all rats. Previously, we captured 61 rats (*R*. *rattus*) in 2012 in the Waikea Forest Reserve in east Hawai‘i Island [19], and only 16.4% of those were visually positive for adult *A*. *cantonensis*. Surveys for *A*. *cantonensis* in rats on the US mainland in Florida report 22.8% (39/171) of *R*. *rattus* were positive [9]. In Louisiana, two *R*. *norvegicus* were found positive and in Oklahoma, one *Sigmodon hispidus* was found positive (total 2.2% or 3/137 brain samples) [22]. However in this study, we observed 72.7% (396/545) of *Rattus* spp. from east Hawaii Island were infected with adult stages of *A*. *cantonensis*, and 90% (490/545) were visually positive for live adult worms, encysted decaying adult worms, and/or L3 larvae embedded in the lung tissue. We also used PCR to test brain tissue of rats which were visually negative (or not completely visually evaluated) for all stages of *A*. *cantonensis*. Including this PCR data, 90% (279/310) of *R*. *rattus*, 99% (198/200) of *R exulans*, and in total, 93.9% (512/545) of all rats from this study were determined to be infected with *A*. *cantonensis*.

Whereas much of the literature on *A*. *cantonensis* has been completed in the L1-L3 stages found in intermediate gastropod hosts, this study visually documents multiple stages within the definitive rat host. We imaged larvated eggs in the lungs and in a female worm (Fig 3a and 3b). L1 larvae were visualized in both rat lungs and feces and were clearly smaller than the L3 larvae (Fig 3c and 3d). Whereas L3 larvae are typically transparent in the gastropod, in the lungs they appear as dark worms due to dark matter in the digestive tract, but not in the esophageal bulb (Fig 3e and 3f). The dark matter may be due to ingested materials or may possibly be refractive granules [20]. Some rats had decaying and/or encysted adult worms in their lung tissues suggesting an older infection, and some concurrently had live adult worms in their lung tissues, indicating multiple infections. Instances were noted where the rats had both adult worms and L3 larvae simultaneously, and some were even positive for live adults, encysted adults, and L3 larvae in the lungs. This diversity in presentation could suggest independent infections occurring serially, concurrently, or as a single infection in which the L3 larvae became trapped in the lungs and never progress to the brain. It is known that not all L3 larvae ingested are successful in reproducing as adults in rats [1], though the limiting mechanism(s) remain(s) a mystery, and it is uncertain in each infection where the ‘lost’ larvae may end up in the tissues. It would be advisable to thoroughly investigate the longevity and impact of larvae that do not reach the brain. In humans or other accidental hosts, especially in the absence of anthelmintic drugs, these potentially long-lived ‘lost’ larvae might continue to cause damage or produce unexpected symptoms over time in unexpected tissues. Whatever the self-limiting mechanism may be present in *A*. *cantonensis*, or *Rattus* spp., or in a dialectic between this parasite and definitive host, it would be useful to define these mechanisms and thus potentially be able to apply the lessons learned in control and treatment efforts.

We found significantly higher rates of infection of adult worms in *R*. *exulans* than in *R*. *rattus*. One possible explanation for this observation could be a greater access to gastropods by *R*. *exulans*. *Rattus exulans* prefers agricultural lands, burrows, and gulches, as compared to *R*. *rattus* (also known as the roof rat) which prefers to nest in high places off the ground [23]. However in our study both species were trapped together, essentially from shared living spaces. All traps were placed on the ground, and both species were caught at the same locations and on the same nights. Also, the semi-slug *P*. *martensi* has shown a propensity for climbing [10] which may mean potential predation on infective intermediate hosts is not necessarily limited to the ground. Another explanation for the higher adult worm infection levels observed in *R*. *exulans* compared to *R*. *rattus* could be related to dietary preference. A previous study conducted on a different island in Hawai‘i observed a greater relative abundance of invertebrates in the diet of *R*. *exulans* (83%) than *R*. *rattus* (40%) [24], although gastropods were not distinguished from other invertebrates.

A significant difference in prevalence of L3 larvae in the lungs was also observed between both rat species, but in this case, the higher levels were in *R*. *rattus*. It is possible that some rat individuals are acquiring L3 larvae outside the trapping areas, as *R*. *rattus* will travel farther in search of food than *R*. *exulans* and may not necessarily feed and nest in the same landscapes [23, 25]. Also *R*. *rattus*, being the larger of the two species, could simply be consuming more infective gastropods than *R*. *exulans*. Regardless of what factors are driving these differences in prevalence between the two host species, why would one host species show higher infection levels in adult worms and another show higher infection levels in L3 larvae? One explanation could be differences in host susceptibility to the parasite, with L3 larvae more successful in completing their lifecycle in certain individuals. While results from our GLM analyses show host species to be a significant predictor of both adult worm and L3 larvae prevalence, host body mass was also identified as a predictor, but only for adult worms and not for the presence of L3 larvae in the lungs (Table 3). Host body mass in wild rats is positively correlated with age [26] and is often used as a proxy for age when comparing groups of individuals [27, 28]. Interestingly, adult worm prevalence in *R*. *rattus* for smaller (younger) individuals was observed at high levels similar to those seen in *R*. *exulans*, but prevalence was significantly lower in the larger (older) *R*. *rattus* individuals (Fig 5). Of note, a previous study in Brazil reported an opposite trend to our findings, with a positive relationship observed between adult *A*. *cantonensis* and host body mass in *R*. *norvegicus* [27]. The authors went on to suggest that these results may be related to a longer period of exposure to infection. So then why would infection levels (both prevalence and intensity) be lower in older *R*. *rattus* in east Hawai‘i? One hypothesis is that these rats’ mature immune systems may be able to clear infections or are somehow able to moderate or regulate the worm burden upon reinfections, with longer living individuals more likely to be reinfected over time. A previous study showed lower adult lungworm burdens in laboratory *R*. *norvegicus* individuals that have been previously infected when compared to those that had not [29]. Additional support for this theory are the opposite trends observed in the mean body masses of *R*. *rattus* individuals found positive and negative for live adult worms (current infections) compared to those for encysted adult worms (previous infections) (Table 2), with the larger/older rats showing more evidence of past infections and less evidence of current infections than smaller/younger rats.

While the presence of encysted adult worms provides clues about previous infections, our observations of granular lung lobes may provide clues about recent infections. Although 100% (197/197) of all rats showing signs of granular lungs were infected with adult worms, the mean body mass for infected *R*. *rattus* individuals was significantly lower in positive vs negative individuals (Table 2). Additionally, granular lungs were observed in only 12% (8/66) of *R*. *rattus* with decaying and/or encysted adult worms, which could suggest the development of granular lungs are not only related to recent infections, but possibly to primary infections (first-time infections). In the rats with granular lungs, it was the upper right lobe which was mostly affected, suggesting that this lobe may serve as a filter for worm passage to the rest of the lung. However the trends observed between body mass and infection levels in *R*. *rattus* were not observed in *R*. *exulans*, the smaller of the two species. Although the lifespan of both host species in the wild is similar, *R*. *rattus* is reported to live slightly longer (mean lifespan of one year or less, with a maximum lifespan of two years) [30] than *R*. *exulans* (mean lifespan of less than on year) [25]. Experimental infections are likely needed to properly determine differences in host susceptibility and parasite survival in definitive rat hosts, and our results emphasize the need for a better understanding of rat lungworm host-parasite dynamics, particularly in Hawai‘i.

If some definitive hosts are indeed able to regulate their parasite burden after multiple infections resulting in the younger, more immunologically naïve individuals contributing disproportionately to the successful transmission of *A*. *cantonensis* in the wild, then this could have major management implications. For example, in a control operation if rat numbers are not reduced to a very low level the remaining rats will rapidly reproduce, and sometimes even exceed their former density [23], potentially leading to a higher ratio of naïve individuals, more capable of successful transmission of L1 larvae to the environment. Additionally, the successful removal of a rat population from an area can free up niche resources within that territory allowing for the potential immigration of rats from outside the area. More research is needed to confirm these scenarios, but if some older rats are indeed regulating their worm burden more effectively than younger rats, then these findings may provide more support for the deworming of certain rat populations in high transmission areas as opposed to the attempted removal of individuals or populations.

While results from our analyses of *R*. *rattus* showed body mass contributed to variation in rat lungworm infection levels at the adult stage, the same was not true for L3 larvae prevalence in the lungs. Study site however, was shown to be a significant factor influencing observed L3 prevalence, but not adult worm prevalence (Table 3). Although more research is needed to explore habitat influences on infection, these findings suggest that while location may be more important for the transmission of infective L3 larvae from intermediate host to definitive host, host characteristics, such as the hosts’ response to previous infections, may be more important in regulating the successful completion of the parasite lifecycle. Studies such as this assist to highlight current gaps in knowledge, that if filled, could allow for a more complete understanding of rat lungworm transmission dynamics, as well as more informed approaches to monitoring infection levels and implementing control measures for wild hosts. Given that east Hawai‘i Island has the highest prevalence of *A*. *cantonensis* in definitive as well as intermediate hosts in the USA, east Hawai‘i Island is the ideal location for conducting future fundamental and applied studies.

## Conclusion

When reporting prevalence of *A*. *cantonensis* infection in rat populations, an important consideration is the careful evaluation of which life cycle stages of the parasite are being sampled. Results from our study show infection with L3 larvae is not necessarily a good indicator of adult worm presence. Although documenting L1 larvae in the feces of a rat may be the only way to truly document completion of the parasite lifecycle in the rat host, the presence of adult worms is a more accurate measure than the presence of L3 larvae. Many studies use PCR to document presence/absence of *A*. *cantonensis* in rats [15, 1, 22, 9], but since this method reports any presence of the parasite DNA, it does not distinguish between life stages. In other words, identifying the infection levels of adult worms in rat populations (i.e. a near completed parasite life cycle) may be more important to understanding the epidemiology of *A*. *cantonensis* in the wild than simply confirming the presence of L3 larvae in the bloodstream or tissues. However even when using the presence of adult worms to report infection levels in rats, our results show that infection can vary greatly by body mass (i.e. age) of the individual, as we report lower infection levels (both prevalence and intensity) in older black rats (*R*. *rattus*); this result in particular holds promise for further research into *Rattus* immune response vis a vis *A*. *cantonensis*. Host body mass and species should always be recorded and presented along with infection level data, especially when attempting to compare levels over time or between different locations.

One of the main pathways for global spread of *A*. *cantonensis* is thought to be through transporting infected rats on voyaging ships [31]. Hilo, HI is a port town, and given the high infection level in local rats, every effort should be made to reduce the spread of *A*. *cantonensis* especially around docks and harbors by implementing deworming protocols in rats, enhancing population control, and/or devising better safeguards for blocking rats from boarding on, or disembarking from ships. The extremely high prevalence of *A*. *cantonensis* observed in *Rattus* spp. in east Hawai‘i Island suggests the potential for human infection is greater than previously thought. Assuming a direct correlation between the introduction and spread of the invasive slug *P*. *martensi* (a highly competent intermediate host for *A*. *cantonensis* and vector for accidental ingestion by humans, pets, and stock animals) and an increase in human cases originating from east Hawai‘i, control measures preventing accidental export of *P*. *martensi* are warranted, especially to warm, subtropical destinations of agricultural significance.

Angiostrongyliasis is currently a serious public health concern in east Hawai‘i. While any gastropod has potential as an intermediate host, *P*. *martensi* is highly effective and has been recently been recorded spreading to another Hawaiian island followed by a corresponding outbreak of human angiostrongyliasis (on Maui). This intermediate host has not yet been reported on the continental US, however, *A*. *cantonensis* is actively gaining entry. As with Zika, dengue, and other emerging tropical diseases, the clear and present danger this complex and difficult-to-eradicate disease constitutes warrants increased measures to control its spread in both gastropods and rodents, thereby protecting human and animal populations, food production, and several sectors of the US economy.

## Supporting information

S1 Video Adult *Angiostrongylus cantonensis* emerging from a ruptured pulmonary artery of a rat. Adult females (larger, helical-striping) and males (smaller) are tightly packed in the pulmonary artery.
(PPTX)

## References

[1] Jarvi SI, Pitt WC, Farias ME, Shiels L, Severino MG, Howe KM, et al Detection of *Angiostrongylus cantonensis* in the blood And peripheral tissues of wild Hawaiian rats (*Rattus rattus*) by a quantitative PCR (qPCR) assay. PLOS ONE 2015: doi: 10.1371/journal.pone.0123064 April 24, 2015. 25910229 10.1371/journal.pone.0123064 PMC4409314

[2] Wang QP, Lai DH, Zhu XU, Chen XG, Lun ZR. Human angiostrongyliasis. *Lancet Infectious Disease* 2008: 8: 621–630. 10.1016/S1473-3099(08)70229-9 18922484

[3] Wang QP, Wu ZD, Wei RL, Owen ZR, Lun ZR. Human *Angiostrongylus cantononsis*: an update. Eur. J Clin Microbiol. Infect. Dis 2012: 31:389–395. doi: 10.1007/s10096-011-1328-5 21725905 10.1007/s10096-011-1328-5

[4] Chen HT. Un nouveau nematode pulmonaire, pulmonema cantonensis, NGN sp. Des rats de Canton. Ann. Parasit. 1935: 13:312.

[5] Rosen L, Chappell R, Laqueur GL, Wallace GD, Weinstein PP. Eosinophilic meningoencephalitis caused by a metastrongylid lungworm of rats. JAMA. 1962: 179: 620–624. 14493905 10.1001/jama.1962.03050080032007

[6] Teem JL, Qvarnstrom Y, Bishop H, da Silva A, Carter J, White-Mclean J, et al The occurrence of the rat lungworm, *Angiostrongylus cantonensis*, in nonindigenous snails in the Gulf of Mexico region of the United States. Hawai‘i J Medicine & Public Health. 2013: 72: (sup. 2) 10–14 PMC3689474 23901374

[7] Chan D, Barratt J, Roberts T, Lee R, Shea M, Marriott D, et al The prevalence of *Angiostrongylus cantonensis/mackerrasae* complex in molluscs from the Sydney Region. PLoS ONE 10, 2015: e0128128. 26000568 10.1371/journal.pone.0128128 PMC4441457

[8] Iwanowicz DD, Sanders LR, Schill WB, Xayavong MV, da Silva AJ, Qvarnstrom Y, et al Spread of the rat lungworm (*Angiostrongylus cantonensis*) in giant African land snails (*Lissachatina fulica*) in Florida, USA. Journal of Wildlife Diseases 2015: 51, 749–753. doi: 10.7589/2014-06-160 25973628 10.7589/2014-06-160

[9] Walden HDS, Slapcinsky JD, Roff S, Calle JM, Goodwin ZD, Stern J, et al Geographic distribution of *Angiostrongylus cantonensis* in wild rats (*Rattus rattus*) and terrestrial snails in Florida, USA PLOS ONE 2017 5 18: https://doi.org/10.1371/journal.pone.0177910 10.1371/journal.pone.0177910 PMC5436845 28542310

[10] Hollingsworth RG, Kaneta RK, Sullivan JJ, Bishop HS, Qvarnstrom Y, da Silva AJ, et al Distribution of *Parmarion* cf. *martensi* (*Pulmonata*: *Helicarionidae*), a new semi-slug pest on Hawai‘i Island, and its potential as a vector for human angiostrongyliasis. Pacific Science. 2007: 61:457–468.

[11] Wilmshursta JM, Hunt TL, Lipoc CP, and Anderson AJ. High-precision radiocarbon dating shows recent and rapid initial human colonization of East Polynesia PNAS 2 1, 2011:| vol. 108 no. 5 418154–1820. www.pnas.org/cgi/doi/10.1073/pnas.1015876108 10.1073/pnas.1015876108 PMC3033267 21187404

[12] Kramer RJ. Hawai‘ian Land Mammals. Charles E. Tuttle Company, Rutland, Vermont 1971.

[13] Atkinson IAE. A reassessment of factors, particularly *Rattus rattus* L., that influenced the decline of endemic forest birds in the Hawai‘ian Islands. Pac Sci 1977; 31(2): 109–133.

[14] Long JL. Introduced mammals of the world: Their history, distribution and influence. CABI Pub, Wal 2003

[15] Qvarnstrom Y, Bishop HS, da Silva A J. Detection of rat lungworm in intermediate, definitive, and paratenic hosts obtained from environmental sources. Hawai’i Journal of Medicine and Public Health. 2013: 72, 63–69. PMC3689491 23901387

[16] Barratt J, Chan D, Sandaradura I, Maliks R, Speilman D, Lee R, et al *Angiostrongylus cantonensis*: a review of its distribution, molecular biology and clinical significance as a human pathogen Parasitology 2016: 143, 1087–111. doi: 10.1017/S0031182016000652 27225800 10.1017/S0031182016000652

[17] Mackerras M, Sandars D. The life history of the rat lungworm, *Angiostrongylus cantonensis* (Chen) (Nematoda: Metastrongylidae). Australian Journal of Zoology 1955: 3, 1–25.

[18] Sikes RS, Gannon WL. Guidelines of the American Society of Mammalogists for the use of wild mammals in research. Journal of Mammalogy. 2011: 92: 235–253. 10.1093/jmammal/gyw078 PMC5909806 29692469

[19] Jarvi SI, Farias MEM, Howe K, Jacquier S, Hollingsworth R, Pitt W. Quantitative PCR estimates *Angiostrongylus cantonensis* infection levels in semi-slugs (*Parmarion martensi*). Molecular and Biochemical Parasitology 2012: 185: 174–176. doi: 10.1016/j.molbiopara.2012.08.002 22902292 10.1016/j.molbiopara.2012.08.002 PMC3753181

[20] Lv S, Zhang Y, Zhang C, Steinmann P, Zhou X, Utzinger J. *Angiostrongylus cantonensis*: morphological and behavioral investigation within the freshwater snail *Pomacea canaliculata*. Parasitol. Res. 2009: doi: 10.1007/s00436-009-1334-z 19172296 10.1007/s00436-009-1334-z

[21] Kim JR, Hayes KA, Yeung NW, Cowie RH. Diverse gastropod hosts of *Angiostrongylus cantonensis*, the rat lungworm, globally and with a focus on the Hawaiian Islands. PLoS ONE. 2014: 9, e94969 doi: 10.1371/journal.pone.0094969 24788772 10.1371/journal.pone.0094969 PMC4008484

[22] York E. M., Creecy J. P., Lord W. D. and Caire W. Geographic range expansion for rat lungworm in North America. Emerging Infectious Diseases. 2015: 21, 1234–1236. doi: 10.3201/eid2107.141980 26079818 10.3201/eid2107.141980 PMC4480392

[23] Marsh R.E. Roof rats. *Prev*. *Control*, 1994: 125–132.

[24] Sugihara RT. Rodent damage research in Hawai‘i: Changing times and priorities. Pages 40–45 in Timm RM and Schmidt RH, eds. Proceedings of the 20th Vertebrate Pest Conference. 2002. University of California, Davis.

[25] Tobin ME. Polynesian Rats. *Prev*. *Control Wildl*. *Damage*, 1994: B121–B124

[26] Hirata DN, Nass RD. Growth and sexual maturation of laboratory-reared, wild *Rattus norvegicus*, *R*. *rattus*, and *R*. *exulans* in Hawaii. J. Mammal. 1974: 55, 472–474. 4833192

[27] Simões RO, Júnior AM, Olifiers N, Garcia JS, Valéria A, Bertolino FA, et al A longitudinal study of *Angiostrongylus cantonensis* in an urban population of *Rattus norvegicus* in Brazil: the influences of seasonality and host features on the pattern of infection. Parasites & Vectors 2014: 7:100. 24612453 10.1186/1756-3305-7-100 PMC3995797

[28] Webster JP, Macdonald DW. Parasites of wild brown rats (*Rattus norvegicus*) on UK farms.*Parasitology*, 1995: 111, 247–255. 7567093 10.1017/s0031182000081804

[29] Au ACS, Ko RC. Changes in worm burden, haematological and serological response in rats after single and multiple *Angiostrongylus cantonensis* infections. Parasitol. Res., 1979: 58, 233–242. 10.1007/BF00933930 452645

[30] Shiels A. B., Pitt W. C., Sugihara R. T., and Witmer G. W.. 2014 Biology and Impacts of Pacific Island Invasive Species. 11. *Rattus rattus*, the Black Rat (Rodentia: Muridae). Pacific Sci. 68: 145–184.)

[31] Kliks MM, Palumbo NE. Eosinophilic meningitis beyond the Pacific Basin: the global dispersal of a peridomestic zoonosis caused by *Angiostrongylus cantonensis*, the nematode lungworm of rats. Soc Sci Med. 1992 1; 34(2):199–212. 1738873 10.1016/0277-9536(92)90097-a

