# Supplementary material for: High prevalence of Angiostrongylus cantonensis (rat lungworm) on eastern Hawai‘i Island: A closer look at life cycle traits and patterns of infection in wild rats (Rattus spp.)
Source: PLoS One. 2017 Dec 18;12(12):e0189458. doi: 10.1371/journal.pone.0189458 (PMC5734720; doi:10.1371/journal.pone.0189458)
Supplement: S1 Video — Adult females (larger, helical-striping) and males (smaller) are tightly packed in the pulmonary artery. (PPTX) [file pone.0189458.s001.pptx]

## Slide 1
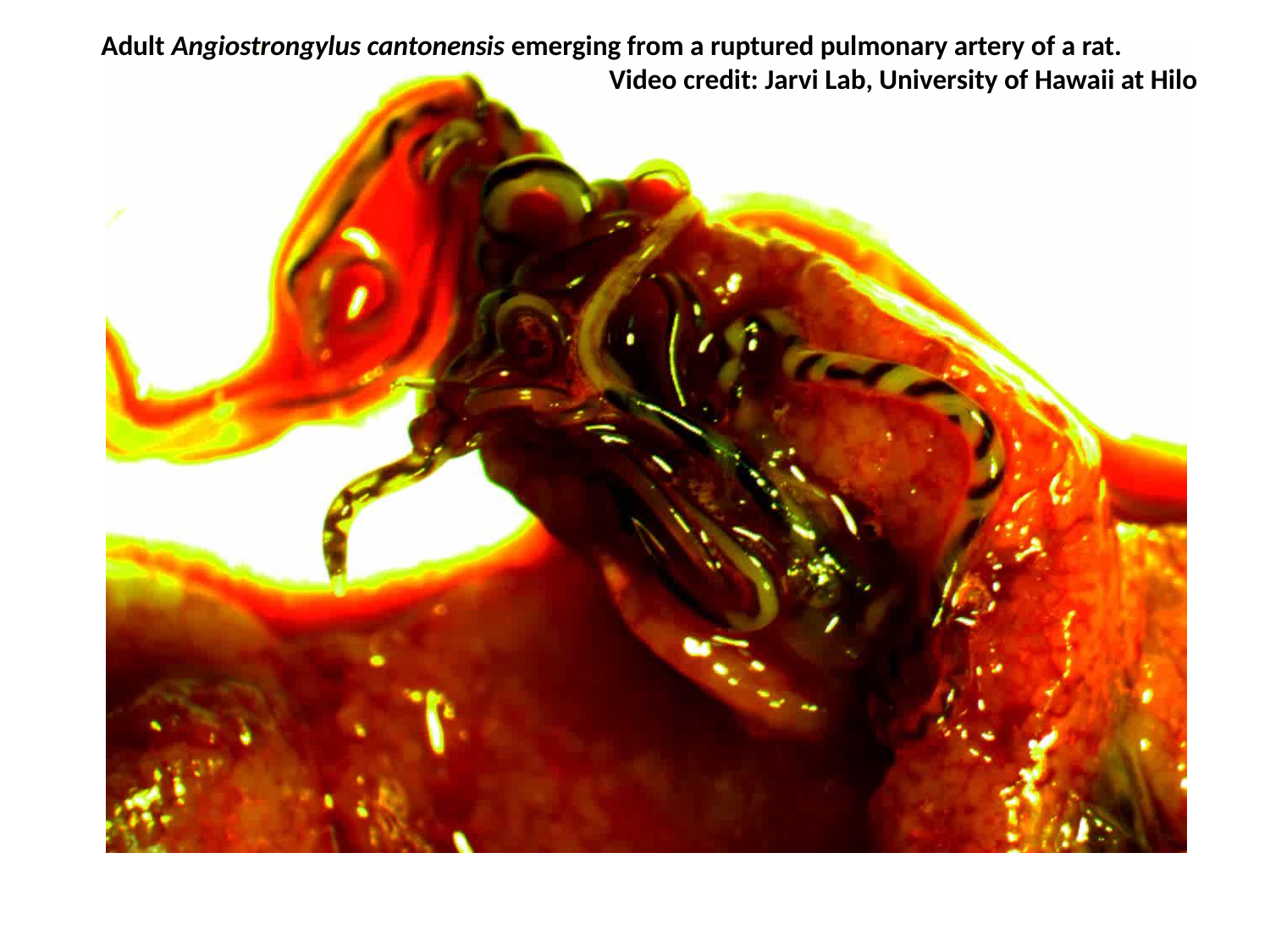

Adult Angiostrongylus cantonensis emerging from a ruptured pulmonary artery of a rat.
				Video credit: Jarvi Lab, University of Hawaii at Hilo
